# Assessment of dental students’ perceptions of facial and smile aesthetics: impact of gender, education level, and family background

**DOI:** 10.1186/s12909-025-07931-z

**Published:** 2025-10-03

**Authors:** Pınar Sesen, Sinem Ok Tokaç, Mehmet Berk Kaffaf, Yesim Sesen Uslu

**Affiliations:** 1 Faculty of Dentistry, Department of Prosthodontics, Istanbul Kent University, İstanbul, Turkey; 2 School of Dental Medicine, Department of Restorative Dentistry, Bahçeşehir University, Istanbul, Turkey

**Keywords:** Gender, Smile aesthetics, Photograph modifications, Perception, Dental students

## Abstract

**Background:**

The aim of the study was to identify whether differences existed in the aesthetic perception of anatomical variations of the face and teeth among dental students, based on factors such as education level, gender, and the presence of a dentist in the family.

**Methods:**

The study was carried out with dental students using the Google Forms platform. A young female model was selected for the survey, with no aesthetic-compromising restorations or pathologies in her maxillary anterior teeth. Standard facial and smile reference photographs were digitally manipulated to alter specific aesthetic features using professional image-editing software (Adobe Photoshop v.20.0.0, Adobe Inc., San Jose, California, USA). Participants evaluated these photographs, focusing on components such as facial symmetry, gingival position, buccal corridor, and occlusal plane angulation. Preclinical and clinical students rated the original and manipulated images on a scale from 1 to 5, with 5 representing the most aesthetically acceptable image and 1 representing the least pleasing one. Statistical analysis comprised Shapiro-Wilk test, Levene test, Mann-Whitney U test.

**Results:**

In the research, 493 students participated, including 240 clinical students; 312 were female, and 89 had a dentist in their family. The overall aesthetic perception score was high (82.63 ± 8.5). Clinical students demonstrated significantly better perceptions of midline diastema, occlusal plane inclination, clinical crown height, and dental modifications compared to preclinical students (*p* < 0.05). Female students scored higher in lower facial height perception than males (*p* = 0.014). The presence of a dentist in the family did not significantly influence aesthetic perception (*p* > 0.05).

**Conclusion:**

Clinical training positively impacted students’ ability to critically analyze aesthetics while maintaining function and naturalness. It can be suggested that, as the academic level increases in dental education, students’ aesthetic judgment skills also improve.

**Supplementary Information:**

The online version contains supplementary material available at 10.1186/s12909-025-07931-z.

## Introduction

The influence of physical appearance on human behavior and social interactions is evident across a wide range of cultures and age demographics [1]. Although the concept of an aesthetically pleasing smile is considered subjective and complex, an ideal smile is often described as one where there is a harmonious relationship between the shape and color of the teeth, and a balanced proportion between the lips and gums [2]. Aesthetics is regarded as a quality that elicits pleasure or satisfaction in individuals, and facial and smile aesthetics emerge from the harmonious interaction of both intraoral and extraoral soft tissues [3, 4].

A smile plays a crucial role in facial expressions and is a key element in social interactions. It is believed that individuals with more attractive smiles tend to have better career prospects and lead more satisfying lives [5]. On the other hand, poor dental aesthetics are linked to a lack of self-confidence, which can impact a person’s social, professional, and academic performance [6].

Although the perception of beauty varies according to geographical, ethnic, cultural, and demographic factors, objective criteria are used as the basis for aesthetic evaluations. In this context, beauty standards derived from various sources, such as measurements specific to certain races and evaluations of individuals generally considered attractive, serve as a guide in achieving an ideal face and smile [3, 7].

Dentofacial aesthetics are evaluated with a more objective approach when assessed by specialist dentists. Specialist dentists take into account guidelines, norms, ideal proportions, and angles based on their professional expertise. In contrast, the perception of beauty among dental students may vary depending on their knowledge levels acquired from different areas of dental education [8]. Dental students are future professionals responsible for oral health [9]. One of the primary objectives of dental education is to train practitioners capable of developing treatment plans based on aesthetic and functional parameters while also respecting patient expectations.

The literature has proven that the perception of aesthetics varies among general dentists, specialists in different dental fields, and individuals in the general population [10–14]. However, studies evaluating the aesthetic understanding of dental students remain limited [3, 15–19].

Therefore, the objective of the current study is to assess dental students’ perceptions regarding various components of facial, smile, and dental aesthetics, using digitally altered images. Furthermore, the study aims to examine whether demographic factors—such as gender, level of clinical training, and the presence of a dentist in the family—have any influence on students’ aesthetic perceptions. The null hypotheses of the study were as follows:

(H_1_) There is no significant difference in students’ perception of aesthetics across the different aesthetic criteria presented in the study.

(H_2_) Students’ aesthetic perceptions are not significantly influenced by demographic characteristics such as gender, clinical education level, or familial dental background.

## Materials&Methods

This cross-sectional survey was approved by the Ethics Committee of the Faculty of Dentistry at Istanbul Kent University (E-10420511-050-29360). Preclinical (second- and third-year) and clinical (fourth- and fifth-year) dentistry students from the Faculties of Dentistry at Istanbul Kent University and Bahçeşehir University were invited to participate. The online questionnaire form was created using Google Forms (Alphabet Co., Mountain View, USA). Prior to participation, informed consent was obtained from all participants, and their involvement was both voluntary and anonymous.

The target population consisted of preclinical and clinical dental students from the two relevant universities in Istanbul. A minimum of 292 participants is estimated to be required to achieve a 95% confidence level with a 5% margin of error, using the following values: *N* = 1200 (population size), Z = 1.96 (Z-score for a 95% confidence interval), *p* = 0.5 (proportion), and e = 0.05 (margin of error). Although the study required 292 participants, the research team attempted to reach the entire population, ultimately contacting 493 students, representing a response rate of 49.3%.

### Photo manipulation

A young female volunteer student was selected as the model for this survey study by four specialists in the fields of Prosthodontics and Restorative Dentistry. This woman was selected due to the absence of restorations or pathological conditions in her maxillary anterior teeth that could affect aesthetics, as well as the overall health of the surrounding tissues. The model exhibited slight dental asymmetry; however, the facial midline aligned with the maxillary dental midline, and the occlusal plane was parallel to both the interpupillary line and the commissural line. Additionally, the model displayed a centered lip line and a convex smile line.

**Fig. 1:**
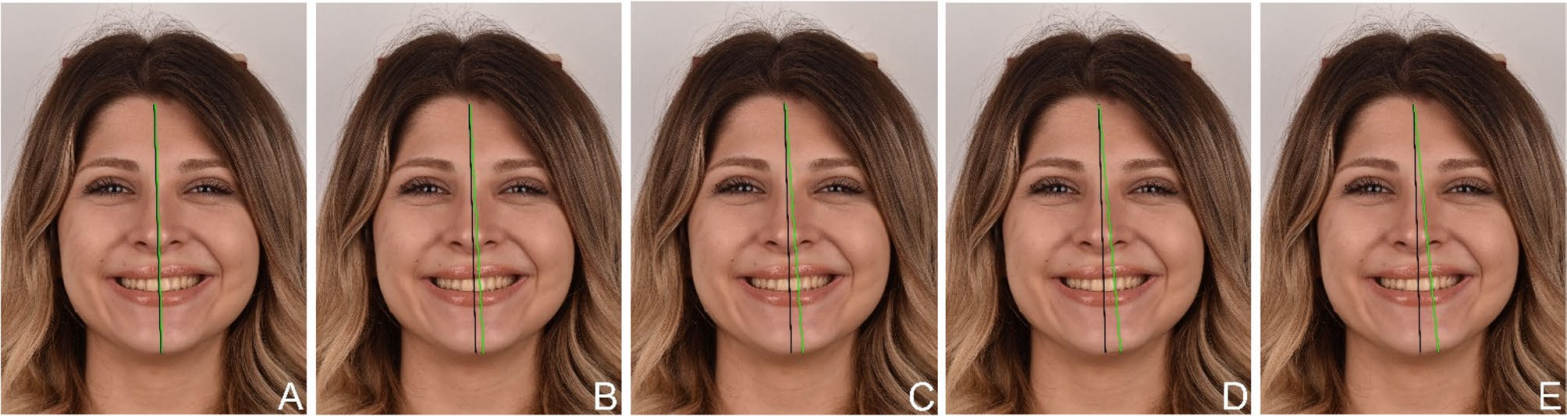
Facial symmetry: Five frontal-view photographs showing progressive degrees of facial asymmetry. (A) No asymmetry (reference image), (B) 4 mm asymmetry, (C) 8 mm asymmetry, (D) 10 mm asymmetry, (E) 14 mm asymmetry

**Fig. 2:**
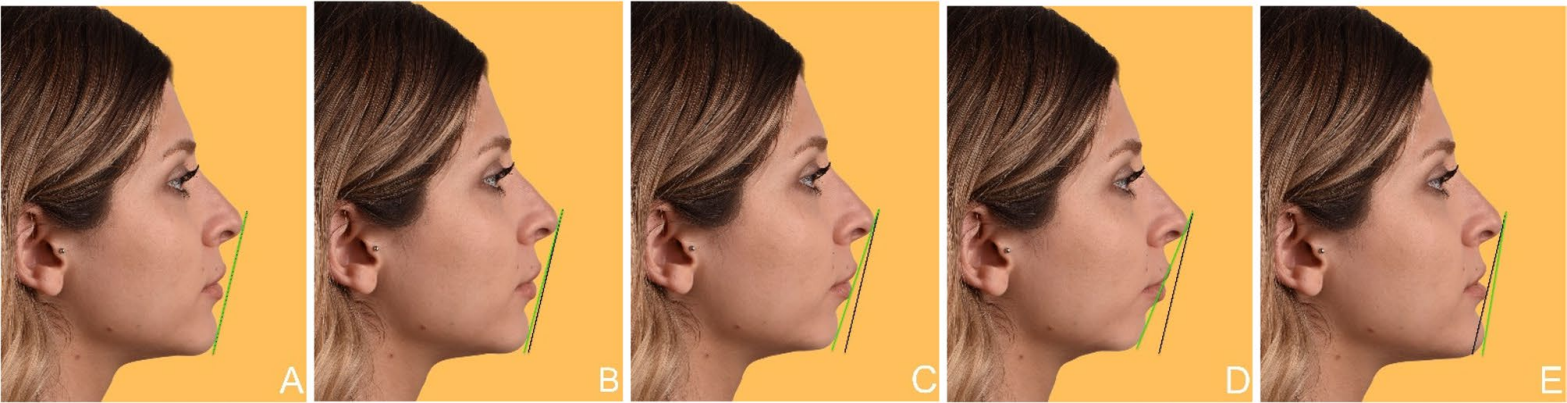
Facial profile modifications: Profile-view photographs showing five variations of facial convexity. (A) Straight profile (reference image), (B) Slightly convex profile, (C) Moderately convex facial profile, (D) Severely convex facial profile, (E) Concave facial profile

**Fig. 3:**
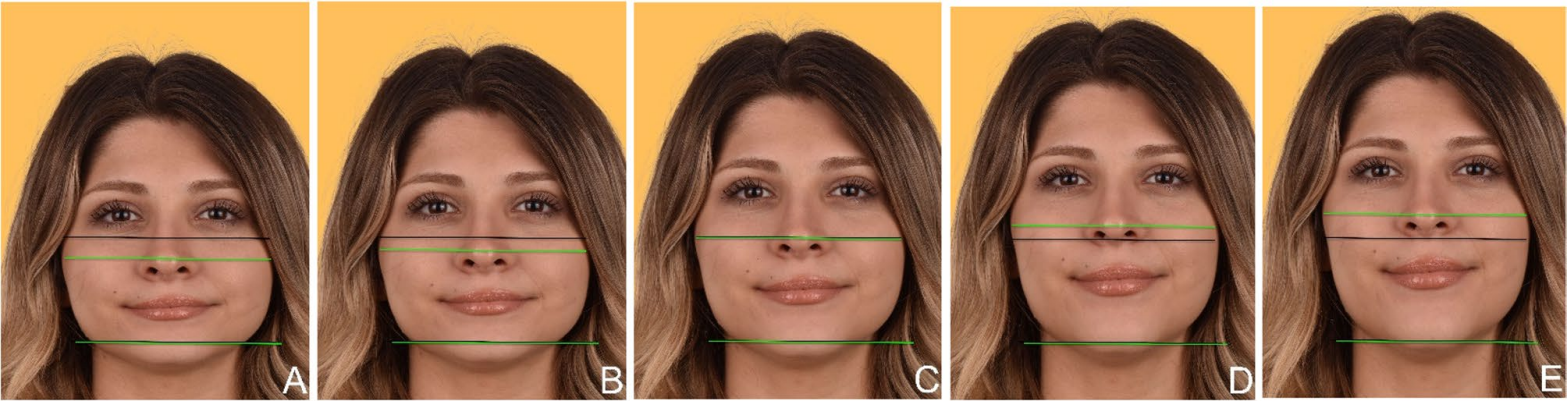
Lower facial height: Frontal-view photographs depicting five variations in lower facial height. (A) Short face (significantly reduced lower facial height), (B) Slightly reduced lower facial height, (C) Average lower facial height (reference image), (D) Slightly increased lower facial height, (E) Long face (significantly increased lower facial height)

**Fig. 4:**
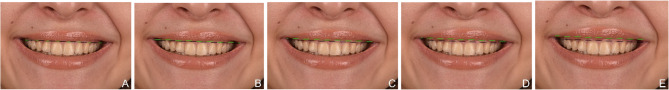
Gingival display: Frontal-view photographs showing increasing levels of maxillary gingival exposure during smiling. (A) Normal gingival display (reference image), (B) 1 mm increased gingival display, (C) 2 mm increased gingival display, (D) 3 mm increased gingival display, (E) 4 mm increased gingival display

**Fig. 5:**
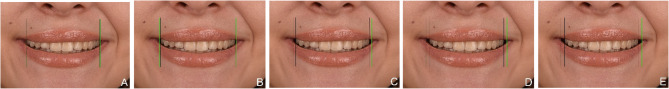
Buccal corridor: Frontal-view smile photographs displaying variations in the width of the buccal corridor. (A) Normal buccal corridor (reference image), (B) Narrow buccal corridor, (C) Very narrow buccal corridor, (D) Wide buccal corridor, (E) Very wide buccal corridor

**Fig. 6:**
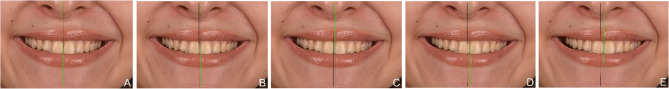
Maxillary midline deviation: Frontal-view photographs showing incremental shifts of the maxillary dental midline relative to the facial midline. (A) No deviation (reference image), (B) 1 mm deviation, (C) 2 mm deviation, (D) 3 mm deviation, (E) 4 mm deviation

**Fig. 7:**
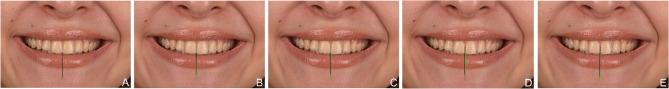
Midline diastema: Frontal-view photographs depicting varying widths of a maxillary midline diastema. (A) No diastema (reference image), (B) 0.5 mm diastema, (C) 1 mm diastema, (D) 1.5 mm diastema, (E) 2 mm diastema. Images demonstrate the progressive increase in interdental spacing between the central incisors for aesthetic assessment

**Fig. 8:**
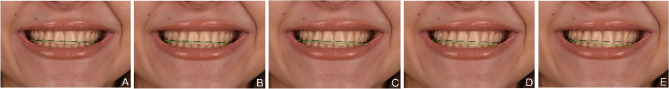
Occlusal plane inclination: Frontal-view smile photographs illustrating increasing degrees of occlusal plane tilt. (A) No inclination (reference image), (B) 1 mm inclination, (C) 2 mm inclination, (D) 3 mm inclination, (E) 4 mm inclination. Images demonstrate progressive inclination of the occlusal plane to assess its impact on smile aesthetics

**Fig. 9:**
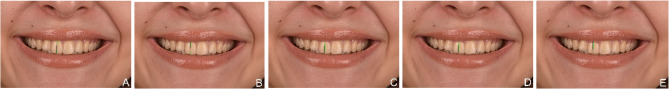
Clinical crown length: Frontal-view photographs displaying progressive shortening of the maxillary right central incisor. (A) No alteration (reference image), (B) 0.5 mm decrease in crown length, (C) 1 mm decrease, (D) 1.5 mm decrease, (E) 2 mm decrease. These images illustrate the esthetic impact of varying reductions in the clinical crown length of a single anterior tooth

**Fig. 10:**
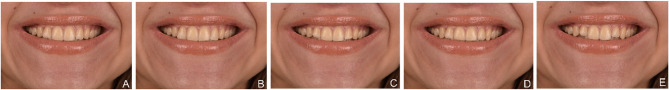
Different dental modifications: Frontal-view photographs showing various alterations in the length and shape of anterior maxillary teeth. (A) Standard smile (reference image), (B) Elongation of both maxillary lateral incisors to match the length of the central incisors, (C) Slope modification of the distal edge of both maxillary lateral incisors, (D) Extension of both canines beyond the length of the maxillary central incisors, (E) Shortening of both maxillary lateral incisors while extending both canines beyond the length of the maxillary central incisors. These modifications were used to evaluate students’ perception of different anterior tooth alterations

Facial and smile reference photographs of a young female patient were digitally manipulated using Adobe Photoshop (v.20.0.0, Adobe Inc., San Jose, California, USA). All photographic modifications were performed using the same image of a volunteer student model who had provided written consent for the use and digital manipulation of her image. To clarify, the same female model was used for all photographic manipulations throughout the study. The digital manipulations included alterations in the chin, facial profile, gingival display, buccal corridor, midline, clinical crown length, occlusal plane inclination, and the position of one or more maxillary teeth. Each standard photograph, along with four modified versions, represented a group for a specific aesthetic feature (Figs. 1, 2, 3, 4, 5, 6, 7, 8, 9 and 10).

## Data collection/online survey

The survey was prepared using Google Forms and consisted of 18 questions divided into three sections. The first section provided a brief explanation of the study and informed participants about the research, asking if they were willing to participate voluntarily. Those who chose not to participate were allowed to exit.

The second section gathered demographic information about the participants, including their gender, age, academic year (current class level), the dental school they were enrolled in, and whether there was a dentist in their family. The third and final section contained questions related to the aesthetic perceptions of the face and teeth, as outlined above.

The aesthetic evaluation questions in the final section, which included photographs, were also presented to the students as a PowerPoint presentation in the classrooms while they completed the online survey. This allowed the participants to view the manipulated images simultaneously during the assessment. The classrooms were large enough to ensure that each participant, regardless of their position, had a clear view of the screen. Additionally, the PowerPoint presentation was projected onto a screen that was sufficiently large to be clearly visible from different angles within the classroom. The PowerPoint presentation consisted of ten slides; each slide represented a group of five numbered photographs, corresponding to a specific aesthetic component as outlined above (Figs. 1, 2, 3, 4, 5, 6, 7, 8, 9 and 10). At the beginning of each slide, the following question was posed: ‘In your opinion, which photograph on this slide has the most aesthetic appearance?

A visual analog scale from 1 to 5 was set up for rating the photographs, where 5 represented the most aesthetically pleasing image and 1 the least pleasing. The scores for each individual component were summed and multiplied by two to calculate an overall aesthetic score, which was then presented on a scale of 100. This method of calculating the overall aesthetic score was conducted in a manner similar to that used in a previous study [3]. The photographs (5 photographs in total) intended for the evaluation of each criterion in the study were presented in a random and varied sequence.

The criteria presented to students through the PowerPoint slides included the following facial components: facial symmetry, chin (Fig. 1), facial profile (Fig. 2), and lower facial height (Fig. 3). The smile components covered were the smile line (gingival display) (Fig. 4) and buccal corridor (Fig. 5). Additionally, the presentation addressed dental components such as midline deviation (Fig. 6), maxillary midline diastema (Fig. 7), occlusal plane inclination (Fig. 8), clinical crown length (gingival component) (Fig. 9), and different dental modifications (Fig. 10).

An English version of the questionnaire is provided as Supplementary File 1 (Appendix 1).

### Statistical analysis

Descriptive statistics for each variable were calculated and presented as the mean ± standard deviation (SD) and median (quartile 1 (25%)–quartile 3 (75%)). Prior to hypothesis testing, the data were examined with a Shapiro-Wilk test for normality and a Levene test for homogeneity of variances, in order to verify the parametric test assumptions. The Mann-Whitney U test was employed to assess the disparities in the ratings of aesthetic components across two groups. A probability value of less than 0.05 was considered statistically significant, unless otherwise noted. SPSS 30 was utilized for statistical analysis. The individual and overall perception of aesthetics were also evaluated using ordinal and linear regressions, respectively, in order to reveal the adjusted effects of the collected variables, including gender, clinical education level, and the presence of a dentist in the family.

## Results

A total of 493 dentistry students participated in the study. The survey was conducted among second, third, fourth, and fifth-year students from two universities. Out of an estimated population of approximately 1,000 students, 493 responses were collected, yielding a participation rate of 49.3%. Based on their level of education, students were categorized into the preclinical group (second- and third-year students, *n* = 253, 51.3%; *n* = 80 males) and the clinical group (fourth- and fifth-year students, *n* = 240, 48.7%; *n* = 100 males) as observed. The number of students with a dentist in their family was 89, accounting for 18.1% of the sample. In this study, 59.6% (*n* = 53) of preclinical students and 40.4% (*n* = 36) of clinical students were found to have a family member who was a dentist. The difference between the two groups (preclinical students, clinical students) was not statistically significant (*p* = 0.086) (Tables 1 and 2).

**Table 1:** Descriptive statistics by demographics

Variable	Category	*n* (%)
Gender	Male	180 (36.5%)
	Female Prefer not to say	312 (63.3%) 1 (0.2%)
Education Status	Preclinical	253 (51.3%)
	Clinical	240 (48.7%)
Any dentist in the family	Yes	89 (18.1%)
	No	404 (81.9%)

(Note: One participant chose not to disclose their gender. This is reflected in the total sample size but excluded from gender-specific analyses.)

**Table 2:** Distribution of preclinical and clinical students by gender and presence of a dentist in the family

	Gender				
	*Male*		*Female*		**P**
Education Status	*n*	*%*	*n*	*%*	
Preclinical Student	80	44,40%	173	55,40%	0,019
Clinical Student	100	55,60%	139	44,60%	

	Any dentist in the family				
	*Yes*		*No*		**P**
Education Status	*n*	*%*	*n*	*%*	
Preclinical Student	53	59,60%	200	49,50%	0,086
Clinical Student	36	40,40%	204	50,50%	

The descriptive statistics and observed differences in the scores obtained from students’ evaluations—categorized by gender, preclinical or clinical student status, and the presence of a dentist in the immediate family—are presented in Tables 3, 4 and 5 respectively.

**Table 3:** Mean and median scores of dental students’ perception of different esthetic components and overall esthetic for the whole sample and by gender

		All sample (*n* = 492)		Males (*n* = 180)		Females (*n* = 312)		
Esthetic components		Mean (SD)	Median (Q1-Q3)	Mean (SD)	Median (Q1-Q3)	Mean (SD)	Median (Q1-Q3)	*P* value
Overall		82.63 (8.5)	84 (78–90)	82.26 (8.4)	82 (78–88)	82.83 (8.57)	84 (76–90)	0,457
Q1	Facial Symmetry	3.7 (1.06)	4 (3–4)	3.82 (1.01)	4 (3–5)	3.63 (1.08)	4 (3–4)	0,071
Q2	Facial Profile	4.24 (0.75)	4 (4–5)	4.24 (0.7)	4 (4–5)	4.24 (0.78)	4 (4–5)	0,727
Q3	Lower Facial Height	4.38 (0.78)	5 (4–5)	4.26 (0.87)	4 (4–5)	4.46 (0.72)	5 (4–5)	0,014
Q4	Gingival Display	4.31 (0.88)	5 (4–5)	4.23 (0.91)	4 (4–5)	4.35 (0.86)	5 (4–5)	0,102
Q5	Buccal corridors	3.5 (0.86)	3 (3–4)	3.51 (0.84)	3 (3–4)	3.49 (0.87)	3 (3–4)	0,589
Q6	Midline deviation	4.02 (0.95)	4 (3–5)	3.94 (1)	4 (3–5)	4.05 (0.92)	4 (3.5-5)	0,31
Q7	Midline diastema	4.68 (0.6)	5 (4–5)	4.64 (0.65)	5 (4–5)	4.7 (0.57)	5 (4–5)	0,546
Q8	Occlusal plane inclination	4.44 (0.82)	5 (4–5)	4.37 (0.89)	5 (4–5)	4.47 (0.78)	5 (4–5)	0,352
Q9	Clinical crown length	4.01 (1.14)	4 (3–5)	4.07 (1.12)	4 (3–5)	3.97 (1.16)	4 (3–5)	0,322
Q10	Different dental modifications	4.05 (1.01)	4 (3–5)	4.04 (1.05)	4 (3–5)	4.05 (1)	4 (3–5)	0,845

**Table 4:** Mean and median scores of dental students’ perception of different esthetic components and overall esthetic by clinical training

		Preclinical (*n* = 253)		Clinical (*n* = 240)		
Esthetic components		Mean (SD)	Median (Q1-Q3)	Mean (SD)	Median (Q1-Q3)	*P* value
Overall		81.74 ± 8.68	82 (76–88)	83.58 ± 8.22	84 (80–90)	0,012
Q1	Facial Symmetry	3.79 ± 0.94	4 (3–4)	3.6 ± 1.16	4 (3–5)	0,159
Q2	Facial Profile	4.23 ± 0.74	4 (4–5)	4.25 ± 0.77	4 (4–5)	0,557
Q3	Lower Facial Height	4.34 ± 0.76	5 (4–5)	4.42 ± 0.8	5 (4–5)	0,093
Q4	Gingival Display	4.32 ± 0.81	4 (4–5)	4.3 ± 0.95	5 (4–5)	0,545
Q5	Buccal corridors	3.53 ± 0.79	3 (3–4)	3.46 ± 0.93	3 (3–4)	0,455
Q6	Midline deviation	3.98 ± 0.84	4 (4–5)	4.05 ± 1.07	4 (3–5)	0,064
Q7	Midline diastema	4.6 ± 0.62	5 (4–5)	4.77 ± 0.57	5 (5–5)	< 0.001
Q8	Occlusal plane inclination	4.32 ± 0.87	5 (4–5)	4.55 ± 0.75	5 (4–5)	< 0.001
Q9	Clinical crown length	3.88 ± 1.14	4 (3–5)	4.15 ± 1.13	5 (4–5)	0,001
Q10	Different dental modifications	3.89 ± 1.02	4 (3–5)	4.22 ± 0.98	5 (4–5)	< 0.001

**Table 5:** Mean and median scores of dental students’ perception of different esthetic components and overall esthetic by presence of a dentist in the family

		Presence of a dentist in the family				
		Yes (n = 89)		No (n = 404)		
Esthetic components		***Mean (SD)***	***Median (Q1-Q3)***	***Mean (SD)***	***Median (Q1-Q3)***	***P value***
Overall		82.7 (7.61)	82 (78–88)	82.62 (8.69)	84 (76–90)	0,777
Q1	Facial Symmetry	3.78 (1.03)	4 (3–5)	3.68 (1.06)	4 (3–4)	0,566
Q2	Facial Profile	4.24 (0.69)	4 (4–5)	4.24 (0.76)	4 (4–5)	0,736
Q3	Lower Facial Height	4.43 (0.69)	5 (4–5)	4.37 (0.8)	5 (4–5)	0,845
Q4	Gingival Display	4.3 (0.95)	5 (4–5)	4.31 (0.87)	5 (4–5)	0,807
Q5	Buccal corridors	3.49 (0.89)	3 (3–4)	3.5 (0.85)	3 (3–4)	0,794
Q6	Midline deviation	4.03 (0.88)	4 (4–5)	4.01 (0.97)	4 (3–5)	0,934
Q7	Midline diastema	4.73 (0.52)	5 (5–5)	4.67 (0.62)	5 (4–5)	0,505
Q8	Occlusal plane inclination	4.48 (0.78)	5 (4–5)	4.43 (0.83)	5 (4–5)	0,577
Q9	Clinical crown length	3.97 (1.14)	4 (3–5)	4.02 (1.14)	4 (3–5)	0,578
Q10	Different dental modifications	3.9 (1.04)	4 (3–5)	4.08 (1)	4 (4–5)	0,107

The overall aesthetic perception among dentistry students was high (82.63 ± 8.5), with the highest ratings observed for midline diastema (4.68 ± 0.6), followed by occlusal plane inclination (4.44 ± 0.82), lower facial height (4.38 ± 0.78), and gingival display (4.31 ± 0.88). These characteristics were also perceived as aesthetically acceptable by more than 75% of the students [Median (Q1–Q3) = 5 (4–5)]. Conversely, the buccal corridor received the lowest scores for aesthetic perception (3.5 ± 0.86), followed by facial symmetry (3.7 ± 1.06). Less than half of the students considered a normal buccal corridor aesthetically acceptable, with a median score [Median (Q1–Q3) = 3 (3–4)]. Notably, 25% of both preclinical and clinical students rated the reference photograph with a score of 3 for buccal corridors aesthetics. When the results were analyzed in detail, 12.3% of preclinical students and 15% of clinical students rated the reference photograph as the most aesthetically acceptable for buccal corridors (Table 6). No statistically significant difference was observed between preclinical and clinical students in their ratings (*p* > 0.05).

**Table 6:** Distribution of scores for reference photographs by educational level

			Education Status					
			Preclinical Student			Clinical Student		
			n	n%	Subgroup (%)	n	n%	Subgroup (%)
Q1	Facial Symmetry	least aesthetically pleasing	6	9,50%	2,40%	17	21,50%	7,10%
		most aesthetically pleasing	57	90,50%	22,50%	62	78,50%	25,80%
Q2	Facial Profile	least aesthetically pleasing	1	1,00%	0,40%	1	1,00%	0,40%
		most aesthetically pleasing	97	99,00%	38,30%	100	99,00%	41,70%
Q3	Lower Facial Height	least aesthetically pleasing	1	0,80%	0,40%	1	0,70%	0,40%
		most aesthetically pleasing	127	99,20%	50,20%	143	99,30%	59,60%
Q4	Gingival Display	least aesthetically pleasing	2	1,60%	0,80%	4	2,90%	1,70%
		most aesthetically pleasing	126	98,40%	49,80%	132	97,10%	55,00%
Q5	Buccal corridors	least aesthetically pleasing	2	6,10%	0,80%	5	12,20%	2,10%
		most aesthetically pleasing	31	93,90%	12,30%	36	87,80%	15,00%
Q6	Midline deviation	least aesthetically pleasing	3	4,10%	1,20%	8	7,00%	3,30%
		most aesthetically pleasing	70	95,90%	27,70%	106	93,00%	44,20%
Q7	Midline diastema	least aesthetically pleasing	0	0,00%	0,00%	0	0,00%	0,00%
		most aesthetically pleasing	166	100,00%	65,60%	198	100,00%	82,50%
Q8	Occlusal plane inclination	least aesthetically pleasing	1	0,70%	0,40%	1	0,60%	0,40%
		most aesthetically pleasing	136	99,30%	53,80%	163	99,40%	67,90%
Q9	Clinical crown length	least aesthetically pleasing	10	9,50%	4,00%	10	7,40%	4,20%
		most aesthetically pleasing	95	90,50%	37,50%	126	92,60%	52,50%
Q10	Different dental modifications	least aesthetically pleasing	4	4,80%	1,60%	4	3,10%	1,70%
		most aesthetically pleasing	80	95,20%	31,60%	124	96,90%	51,70%

A significant gender difference was observed in the perception of lower facial height, with female students assigning higher scores compared to their male counterparts (*p* = 0.014). This finding was further supported by ordinal regression analysis, which confirmed that female students were significantly more likely to assign higher aesthetic scores for lower facial height than males (β = −0.500, *p* = 0.006), (Table 3).

The results illustrating the impact of clinical training, or in other words, the level of dental education, on the perception of facial and dental aesthetics are presented in Table 4. Clinical students perceived overall esthetic components significantly higher than preclinical students (Median [Q1-Q3]: 84 [80–90] vs. 82 [76–88]; *p* = 0.012) (Table 4). Ordinal regression analysis further confirmed that clinical education level was a significant predictor of higher aesthetic perception scores (β = −1.916, *p* = 0.013), with clinical students demonstrating significantly higher ratings compared to preclinical students (Table 7).

**Table 7:** Independent determinants of dental students’ perception of different esthetic components as shown by ordinal regression analyses

	Parameter	Estimate	Std. Error	P value	95% Confidence Interval	
					Lower Bound	Upper Bound
Q1	Male (Ref: Female)	0,327	0,172	0,057	−0,01	0,663
	Preclinical (Ref: Clinical)	0,245	0,165	0,139	−0,079	0,569
	Dentist in the family: Yes (Ref: No)	0,101	0,215	0,64	−0,32	0,521
Q2	Male (Ref: Female)	−0,072	0,179	0,687	−0,422	0,278
	Preclinical (Ref: Clinical)	−0,118	0,173	0,493	−0,457	0,22
	Dentist in the family: Yes (Ref: No)	−0,025	0,224	0,91	−0,464	0,414
Q3	Male (Ref: Female)	−0,5	0,182	0,006	−0,856	−0,143
	Preclinical (Ref: Clinical)	−0,373	0,178	0,036	−0,722	−0,025
	Dentist in the family: Yes (Ref: No)	0,143	0,231	0,536	−0,31	0,596
Q4	Male (Ref: Female)	−0,308	0,179	0,085	−0,66	0,043
	Preclinical (Ref: Clinical)	−0,134	0,174	0,443	−0,475	0,208
	Dentist in the family: Yes (Ref: No)	0,074	0,227	0,744	−0,37	0,518
Q5	Male (Ref: Female)	0,107	0,176	0,544	−0,238	0,451
	Preclinical (Ref: Clinical)	0,141	0,17	0,406	−0,192	0,474
	Dentist in the family: Yes (Ref: No)	0,016	0,22	0,941	−0,415	0,448
Q6	Male (Ref: Female)	−0,208	0,173	0,229	−0,548	0,131
	Preclinical (Ref: Clinical)	−0,322	0,168	0,055	−0,652	0,007
	Dentist in the family: Yes (Ref: No)	0,023	0,217	0,917	−0,403	0,449
Q7	Male (Ref: Female)	−0,224	0,216	0,298	−0,647	0,198
	Preclinical (Ref: Clinical)	−0,888	0,216	< 0.001	−1,312	−0,464
	Dentist in the family: Yes (Ref: No)	0,27	0,279	0,333	−0,276	0,817
Q8	Male (Ref: Female)	−0,241	0,188	0,201	−0,609	0,128
	Preclinical (Ref: Clinical)	−0,636	0,185	< 0.001	−0,998	−0,274
	Dentist in the family: Yes (Ref: No)	0,218	0,24	0,365	−0,253	0,689
Q9	Male (Ref: Female)	0,125	0,175	0,474	−0,218	0,468
	Preclinical (Ref: Clinical)	−0,519	0,17	0,002	−0,851	−0,187
	Dentist in the family: Yes (Ref: No)	−0,084	0,217	0,699	−0,51	0,342
Q10	Male (Ref: Female)	−0,003	0,174	0,987	−0,345	0,339
	Preclinical (Ref: Clinical)	−0,676	0,17	< 0.001	−1,01	−0,343
	Dentist in the family: Yes (Ref: No)	−0,306	0,216	0,157	−0,73	0,117
Overall	Male (Ref: Female)	−0,797	0,799	0,319	−2,367	0,772
	Preclinical (Ref: Clinical)	−1,916	0,772	0,013	−3,432	−0,399
	Dentist in the family: Yes (Ref: No)	0,265	1,001	0,791	−1,703	2,233

This notable difference encompassed the criteria of midline diastema, occlusal plane inclination, clinical crown length, and different dental modifications. Clinical students perceived midline diastema significantly higher than preclinical students (Median [Q1-Q3]: 5 [5, 5] vs. 5 [4, 5]; *p* < 0.001). A significant difference was found between clinical and preclinical students in the perception of occlusal plane inclination, with clinical students assigning higher ratings (Median [Q1-Q3]: 5 [4, 5] vs. 5 [4, 5]; *p* < 0.001). Clinical students also rated clinical crown length higher compared to preclinical students (Median [Q1-Q3]: 5 [4, 5] vs. 4 [3–5]; *p* = 0.001). Clinical students (4.22 ± 0.98) Median [Q1–Q3]: 5 [4, 5] demonstrated a more accurate perception of different dental modifications compared to preclinical students (3.89 ± 1.02) Median [Q1–Q3]: 4 [3–5]; *p* < 0.001.

The presence of a dentist in the family did not result in a significant difference in students’ aesthetic perception *p*>0.05 (Table 5).

## Discussion

Many scientific studies have investigated smile aesthetics, often using digitally altered images and photographs for evaluation [3, 14, 16, 20–23]. The present study evaluated the perception of facial, dental, and smile aesthetic variations among dentistry students and examined whether these perceptions differed based on education level, gender, and the presence of a dentist in the family. In the present study, variations in the perception of aesthetic criteria among students were observed, indicating that not all criteria were evaluated similarly. Therefore, the first null hypothesis (H1) was rejected. Regarding the second null hypothesis (H2), gender influenced the perception of lower facial height, and significant differences were found between preclinical and clinical students in their evaluation of midline diastema, occlusal plane inclination, clinical crown length, and various dental modifications. However, the presence of a dentist in the family had no significant effect. Accordingly, the second hypothesis was partially rejected.

The attractiveness of the face directly influences the appeal of a smile. Aesthetic evaluations depend not only on dental features but also on facial aesthetics. However, the use of a full-face smile as a gold standard for dentofacial aesthetic analyses remains debated. It is important to consider that factors such as soft tissue, buccal corridors, and gingival display, along with intraoral features, also contribute to aesthetic appeal [24]. Intraoral images allow observers to focus more on dental elements (e.g., smile, gingiva, color, and dental components) by reducing distractions from other aspects [21]. Therefore, this study employed both full-face and intraoral photographs to assess facial and smile aesthetics.

Kovačić et al. evaluated how education level and gender influenced dental students’ perceptions of various aesthetic criteria, including tooth shade. They found that clinical students were more critical and rated modifications, particularly lighter tooth shades, with lower scores compared to preclinical students [16]. In present study, however, no manipulations were made to tooth color to ensure the standardization of the photographs and to allow for the evaluation of only the specified criteria. Aesthetic assessments were conducted using standardized reference photographs of a single model, with variables such as tooth color kept constant. This approach ensured that the potential impact of color changes on aesthetic perception was eliminated, providing a controlled basis for evaluating other aesthetic parameters.

In the literature, the impact of gender on dentofacial aesthetic perception has been reported to be largely culture-dependent. Abu Alhaija et al. also found that gender influences aesthetic perception, with women being more sensitive to gingival display and midline diastema than men [25]. However, Moore et al. and Ioi et al. reported that men and women assess smile attractiveness similar manner [26, 27]. In a literature review evaluating dental students’ perceptions of dental aesthetics, it was reported that female students tend to have a more critical self-perception regarding the characteristics of an ideal smile. Furthermore, it is important to consider that dental students’ aesthetic perceptions are also influenced by their level of education and cultural factors [9].

In this study, a statistically significant gender difference was observed only in the lower facial height criterion, with female students demonstrating a higher aesthetic perception compared to their male counterparts (*p* = 0.014). The majority of participating dentistry students evaluated the reference photo as the most aesthetically pleasing and acceptable face. Among the evaluated photos, those with increased lower facial height received the lowest aesthetic scores. Additionally, lower facial height was one of the criteria where students exhibited the highest aesthetic perception. There are very few studies in the literature that examine how dentistry students evaluate lower facial height. In the study by Alhammadi et al., the majority of students considered slightly reduced, slightly increased, or average vertical facial proportions as the most aesthetically pleasing, which differs from the findings of the present study [3]. Unlike Alhammadi et al., both extreme variations—particularly increased lower facial height—were perceived less favorably, while the unaltered reference image was most preferred. These differences may be attributed to cultural, demographic, or methodological variations between the two studies.

In this study, the overall aesthetic perception among dental students was found to be high (82.63 ± 8.5). This value was higher than those reported by Omar and Tai [14] and similar to the findings of Alhammadi et al. [3]. The lowest perception score was observed for the buccal corridor, followed by facial symmetry. Less than 25% of dental students (12.3% of preclinical and 15% of clinical students, score 5, Table 7) correctly identified the standards for the buccal corridor. Detailed analysis of the results revealed that a reduced and narrow buccal corridor was perceived as more aesthetic in this study. Despite the challenges faced by the students, the reference photograph for this criterion was rated as the least aesthetic by only 0.8% of preclinical students and 2.1% of clinical students (score 1, Table 7). Similarly, a previous survey-based study also identified the buccal corridor as the lowest-rated aesthetic evaluation criterion [3]. Another study conducted in Japan reported that dental students and orthodontists showed similar perceptions of the buccal corridor [27]. Although our study did not include orthodontists, this literature finding highlights how the perception of this feature may vary across different educational or cultural contexts. The fact that Japanese dental students evaluated the buccal corridor similarly to orthodontists may be attributed to the superior quality of undergraduate dental education in Japan. The buccal corridor has been reported as a challenging criterion to assess, even for dental specialists [28], and it plays a significant role in overall smile aesthetics [29]. Furthermore, the lack of a significant difference between clinical and preclinical students in evaluating the buccal corridor highlights the need for improved education and training on this complex aesthetic parameter.

The second lowest aesthetic perception criterion, facial symmetry, was correctly identified by less than half of the students. Facial symmetry, defined as the balanced alignment of facial features relative to the mid-sagittal plane, is a fundamental aspect of facial beauty and attractiveness [30]. Recognizing facial symmetry is crucial for clinicians in diagnosis and treatment planning, as well as for patients in achieving desired aesthetic outcomes. In our study, 22.5% of preclinical students and 25.8% of clinical students (Table 7) perceived the reference photograph as aesthetic, with no significant difference between the two groups. Additionally, this criterion was not influenced by gender. Notably, facial symmetry was the criterion with the highest rate of reference image being rated as the least esthetic among clinical students, at 7.10% (Table 11), indicating that some participants may have misinterpreted the ideal image in this category. Although one study reported that the vast majority of dental students could detect facial asymmetries up to 2 mm [3], another study suggested that deviations of up to 5 mm may still be perceived as within the normal range [31]. In the present study, facial asymmetry of up to 4 mm was also perceived as aesthetic. Discrepancies between studies may stem from methodological variations and differences in evaluator populations.

The facial profile, often used by orthodontists to clinically assess facial harmony, is a significant factor in determining facial attractiveness [3]. In this study, students found straight, slightly convex, and then concave profiles to be the most aesthetic, in that order. Among the participants, 38.3% of preclinical students and 41.7% of clinical students (Table 7) rated the reference photograph as aesthetic, with no significant difference between the groups. Similar findings have been reported in previous studies, where straight and slightly convex profiles were generally perceived as more attractive [32]. In the study by Torul et al., no significant difference was found between preclinical and clinical students; however, unlike our findings, they identified a difference between male and female students [33].

The harmony between clinical crown length and gingival display during a smile plays a critical role in aesthetics and holds significant clinical importance in smile design planning. Excessive gingival display, commonly referred to as a gummy smile (> 3 mm), is often considered unaesthetic [34]. In this study, the gingival display criterion was among the parameters where dental students demonstrated a high level of aesthetic perception. Notably, more than 75% of students accurately perceived the reference photograph as aesthetic, with no significant differences between preclinical and clinical students or between male and female participants.

Consistent with these findings, it was reported that the majority of dental students, regardless of gender, were able to recognize normal gingival display or an increase of up to 1 mm [3]. Ayyildiz et al. reported significant differences between preclinical and clinical students in their evaluation of gingival smiles as part of facial aesthetics [34]. Similarly, Čalušić Šarac et al. found that expert dentists, doctors, and clinical dental students assessed gingival asymmetries more critically, noticing even minor variations, whereas preclinical students and laypeople could only detect gingival asymmetries of 2 mm or more [35]. The consistent and high level of perception observed for this criterion may suggest that the dental curriculum effectively promotes awareness of gingival aesthetics.

In the present study, a midline deviation of up to 1 mm was perceived by more than 75% of dental students. While 27.7% of preclinical students and 44.2% of clinical students rated the reference photograph as the most aesthetic, this difference was not statistically significant (Table 7). Consistent with the findings of this study, a previous study also reported that more than 75% of students perceived midline deviations of up to 1 mm as acceptable [3]. However, in that study, it was noted that this criterion was more accurately identified by male students, differing from the present findings [3]. In another study, it was reported that midline deviations of 2 mm or less were considered acceptable by females, whereas deviations of 3 mm or more were deemed acceptable by males [36]. Vertically, the facial midline is determined by two anatomical reference points: the nasion and the philtrum. Aesthetically, the dental midline is expected to align with the facial midline. According to the literature, the average acceptable deviation is generally reported to be 2.38 mm [37]. However, Rodrigues et al. found no difference in the perception of an ideal smile and a midline deviation of up to 3 mm [7]. Tahir et al. reported that dental professionals were able to detect midline deviations exceeding 2mm, whereas laypersons did not notice deviations up to 4 mm [22]. In this study, detailed analysis of the data revealed that more than half of the students identified a 4 mm deviation as the least aesthetically acceptable.

In this study, clinical students exhibited significantly higher aesthetic perception than preclinical students in detecting changes in Q7 midline diastema, Q8 occlusal plane inclination, Q9 clinical crown length, and Q10 different dental modifications, with statistically significant differences. Similar to the present study, previous research has reported that clinical students are more successful than preclinical students in detecting minimal changes and that aesthetic awareness increases with higher levels of education [3, 38]. A previous study conducted among dentists, dental students, and laypersons demonstrated the impact of education on aesthetic perception. While a direct comparison between preclinical and clinical students was not made, the findings highlighted the influence of educational level on aesthetic awareness [39]. A review study indicated that the examined studies consistently reported an improvement in students’ ability to analyze aesthetics as they gain more knowledge and clinical experience [9]. This may be due to the fact that students in their early years primarily focus on general medical courses, and dental courses are limited during the early years of their curriculum. In contrast, clinical dental students in higher years are exposed to more dental courses and clinical practice; therefore, they may have become more critical of small deviations from an ideal smile.

Among the participants, 65.6% of preclinical students and 82.5% of clinical students rated the reference photograph for the midline diastema criterion as the most aesthetic. This criterion was identified as having the highest aesthetic perception in our study. Noureddine et al. evaluated both multiple diastemas of the anterior teeth and midline diastema, concluding that the midline diastema was perceived as the least aesthetic [40]. It has been suggested that its negative impact is influenced by the width of the diastema [40]. In the study by Kovacic et al., while clinical students assigned lower scores to all modified photographs compared to preclinical students, the lowest ratings in both groups were given to the lightest shades and the midline diastema [16]. In this aspect, the related studies were similar to the present study.

For the occlusal plane inclination criterion, the reference photograph was rated as the most aesthetic by 53.8% of preclinical students and 67.9% of clinical students, with a statistically significant difference between the groups. In the present study, the occlusal plane inclination was identified as the second-highest criterion in terms of aesthetic perception. Revilla-León et al. determined that occlusal plane inclinations of 0 and 1 degree showed no perceptible differences in smile evaluation among dentists, dental students, and laypersons [41]. In their study, the dentist and dental student groups gradually assigned lower scores as the inclination increased, while laypersons only gave lower scores when the occlusal plane inclination reached 3 degrees or more [41]. In the current study, almost 45% of the students rated the 4 mm inclination as the least aesthetic.

In this study, the crown length of the maxillary right central incisor was altered in increments of 0.5 mm, up to 2 mm, for each reference photograph. The reference photograph was rated as the most aesthetic by 37.5% of preclinical students and 52.5% of clinical students, with a statistically significant difference between the groups. In a study, the perceptions of dental professionals and laypersons regarding bilateral crown length alterations were evaluated, and the threshold for unattractiveness was reported as 1.0 mm for orthodontists, 1.5 mm for general dentists, and 2.0 mm for laypersons [13]. In the current study, approximately 35% of the students scored a reduction of up to 2 mm in the right central incisor as the least aesthetic appearance. Interestingly, among all the esthetic parameters evaluated, clinical crown length was the only criterion for which preclinical students most frequently identified the reference image as the least esthetic, with 4% rating it as the least attractive (Table 11). Overall, the rate at which preclinical students rated the reference image as the least esthetic was low for other criteria. This finding suggests that crown length discrepancies may be a more ambiguous or unfamiliar esthetic factor for students at earlier stages of dental education, likely due to their limited clinical experience. It also indicates that even mild, unilateral reductions in crown lengthmay not always be perceived as esthetically unfavorable by inexperienced students.

In the current study, the final question of the survey focused on various dental modifications. Modifications included the elongation of both maxillary lateral incisors to match the central incisors, slope alteration of the distal edges of maxillary lateral incisors, extension of both canines beyond the length of the central incisors, and shortening of the lateral incisors while extending the canines. The modification that students found the least aesthetic was the shortening of the lateral incisors while extending the canines, with approximately 35% of students selecting this option. Schwefer et al. also found in their study that the condition where the canines were longer than the central incisors was rated as the least aesthetic [21]. This finding is consistent with the results of our study. The reference photograph was rated as the most aesthetic by 31.6% of preclinical students and 51.7% of clinical students, with a statistically significant difference between the groups. Studies specifically assessing the perception of various dental modifications among preclinical and clinical students are limited in the existing literature. Kovacic et al. conducted a study evaluating the perception of various dental modifications [16]. Similar to our findings, they observed differences in the evaluation of these dental modifications between preclinical and clinical students. Clinical students were more proficient in evaluating dental modifications [16]. This question aimed to compare various dental modifications ranging from the most to the least esthetic, as perceived by students. These findings support the clinical relevance of assessing combined dental modifications in a single visual, as clear preference patterns still emerged.

In this study, having a dentist in the family did not have a significant impact on the aesthetic perception of dental students. The targeted academic education level, however, was more effective in acquiring the knowledge and skills necessary to notice changes in smile aesthetics criteria.

It is important to address the few limitations of this study. It was observed that the majority of students participating in this study were female. This imbalance reflects the demographic structure of the universities involved in the study, which have a higher enrollment of female students. Factors such as students’ socioeconomic status, the influence of social media, and fashion trends were not included in this study. Furthermore, the researchers focused on specific smile aesthetics criteria that they deemed significant in the perception of an aesthetically pleasing smile. Although the survey was completed online, the images were presented in a classroom setting to ensure standardization and equal visual conditions. However, this setting may have introduced response biases. Social desirability bias and the Hawthorne effect [42] could have influenced students’ answers due to the presence of peers and the structured environment. Future studies should address a broader range of variables and include analyses involving male and female subjects from diverse ethnic backgrounds. Additionally, future studies should consider including participants from different universities and regions to better reflect the influence of cultural and geographic diversity on aesthetic perception.

## Conclusion

Within the limitations of this study, the following conclusions can be drawn:

- Targeted academic education effectively influences aesthetic perception.
- Clinical students exhibited greater accuracy in perceiving midline diastema, occlusal plane inclination, clinical crown length, and various dental modifications compared to preclinical students.
- The presence of a dentist in the family did not have a significant impact on aesthetic perception. This also suggests that structured dental education may be more impactful than personal exposure through familial background.
- Female students demonstrated a more accurate perception of lower facial height compared to their male counterparts, suggesting a gender-related difference in aesthetic evaluation for this specific parameter.
- The findings suggest that awareness of complex and subtle aspects of dentofacial aesthetics, such as the buccal corridor and facial symmetry, should be further emphasized and strengthened within undergraduate dental education programs.

## Supplementary Information

Supplementary Material 1.

Supplementary Material 2.

Supplementary Material 3.

Supplementary Material 4.

## Data Availability

The datasets used and/or analyzed during the current study are available from the corresponding author on reasonable request.

## Abbreviations

VAS: Visual Analog Scale
SD: Standard Deviation
CI: Confidence Interval
SPSS: Statistical Package for the Social Sciences
p: Probability Value
β: Beta Coefficient
OR: Odds Ratio
Q1: Q3–First and Third Quartiles
ANOVA: Analysis of Variance
Mann: Whitney U test–Non–parametric statistical test
Shapiro: Wilk test–Normality test
Levene test: Homogeneity of variances test
