# Supplementary material for: Assessment of dental students’ perceptions of facial and smile aesthetics: impact of gender, education level, and family background
Source: BMC Med Educ. 2025 Oct 3;25:1350. doi: 10.1186/s12909-025-07931-z (PMC12495680; doi:10.1186/s12909-025-07931-z)
Supplement: Supplementary file 1 — Supplementary Material 1. [file 12909_2025_7931_MOESM1_ESM.pdf]

Evaluation of Aesthetic Perception of Dentistry Students

Dear Colleague,

This study aims to evaluate dentistry students' perceptions of face, smile and dental aesthetic components and whether this perception is affected by gender and clinical education level.

This study is carried out by Asst. Prof Pınar ŞEŞEN, Asst. Prof. Mehmet Berk KAFFAF, Asst. Prof. Sinem Ok TOKAÇ and Assoc. Prof. Yeşim ŞEŞEN USLU.
After you fill out this survey form, your answers and personal information will remain confidential and will only be used for scientific purposes for this study. The time to fill out the survey is approximately 15 minutes. Students at preclinical education level and clinical education level actively caring for patients are expected to participate in this study.

For your questions, valuable contributions and suggestions, you can contact us via pinar.sesen@kent.edu.tr or 0546 528 5098.

Thank you for your participation.

* indicates a mandatory question

1. If you want to continue with the survey, select 'yes'. (This question will be used as consent.) *

Yalnızca bir şıkkı işaretleyin.

- Yes
- No

DEMOGRAPHIC INFORMATION

2. Please indicate your sex *

Mark only one option

- Female
- Male

3. Write your date of birth. *

4. Please indicate your educational status. *

Mark only one option

- Preclinical
- 4th year clinical student
- 5th year clinical student

5. The university you are currently studying at *

Mark only one option

- State University
- Private (Foundation) University

6. Is there a dentist in your family? *

Mark only one option

- Yes
- No

AESTHETIC ANALYSIS QUESTIONS

Dear students, please evaluate each question according to:
"From your point of view, which photo is the most aesthetic for each question?"

5= Very beautiful
4=Beautiful
3= Undecided
2= Ugly
1= Very ugly

Photo-1

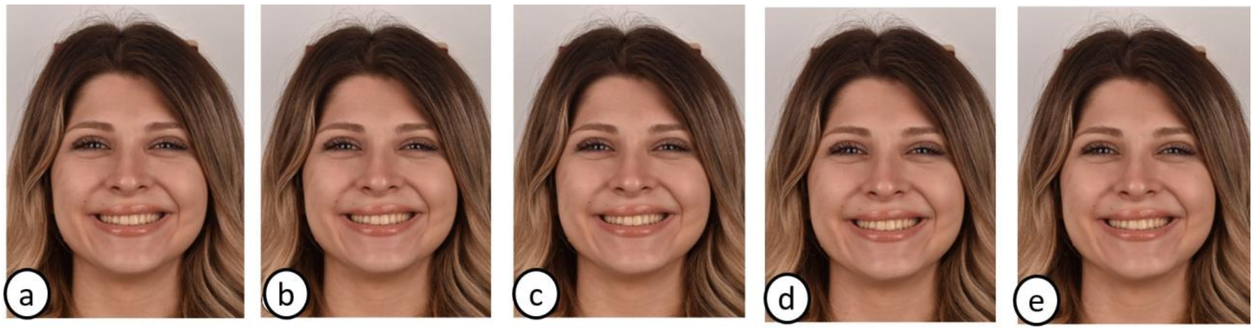

7. Photo-1 *

Mark only one option for each line.

	Very beautiful	Beautiful	Undecided	Ugly	Very ugly
a					
b					
c					
d					
e					

Photo-2

Photo 2

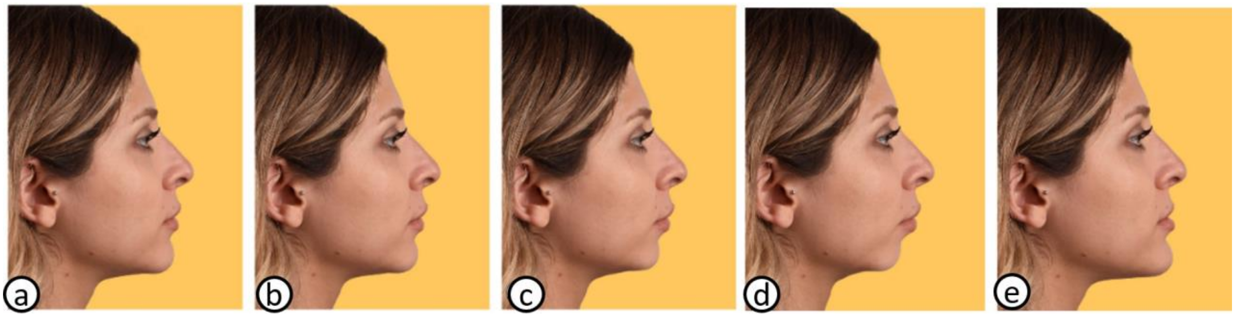

8. Photo-2 *

Mark only one option for each line.

	Very beautiful	Beautiful	Undecided	Ugly	Very ugly
a					
b					
c					
d					
e					

Photo-3

Photo 3

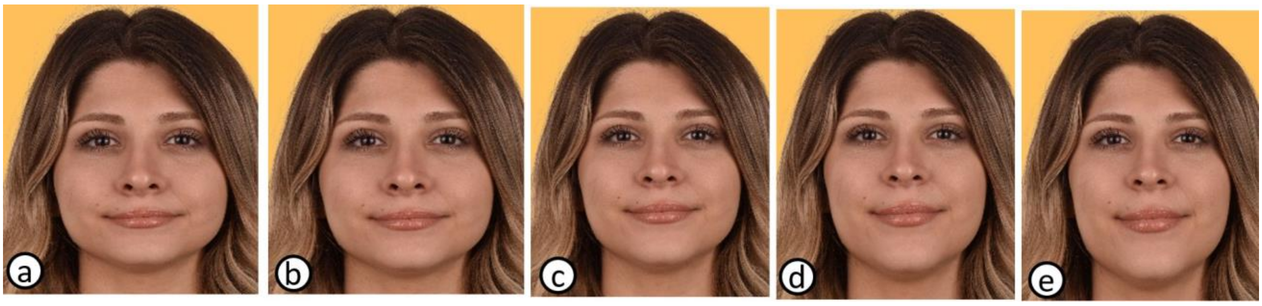

9. Photo-3 *

Mark only one option for each line.

	Very beautiful	Beautiful	Undecided	Ugly	Very ugly
a					
b					
c					
d					
e					

Photo-4

Photo 4

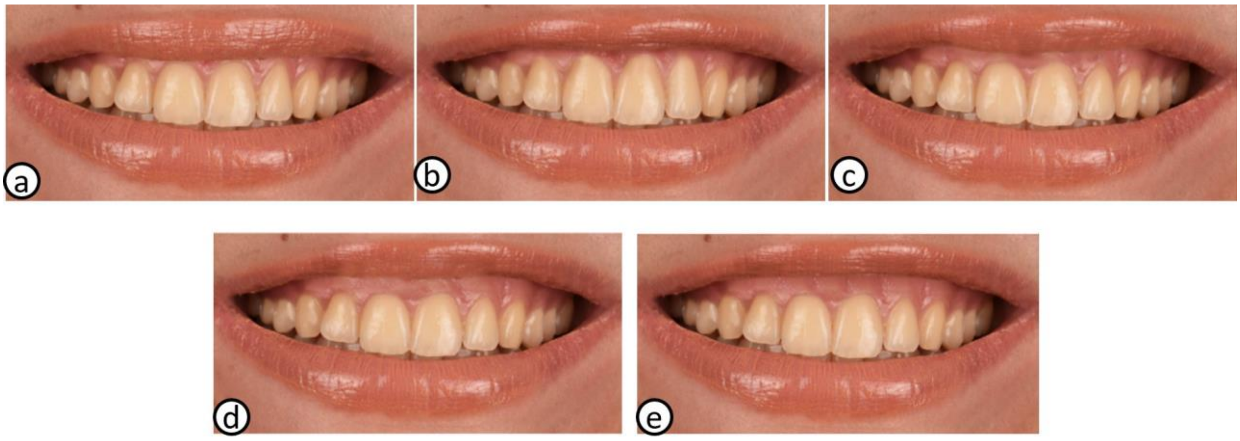

10. Photo-4 *

Mark only one option for each line.

	Very beautiful	Beautiful	Undecided	Ugly	Very ugly
a					
b					
c					
d					
e					

Photo-5

Photo 5

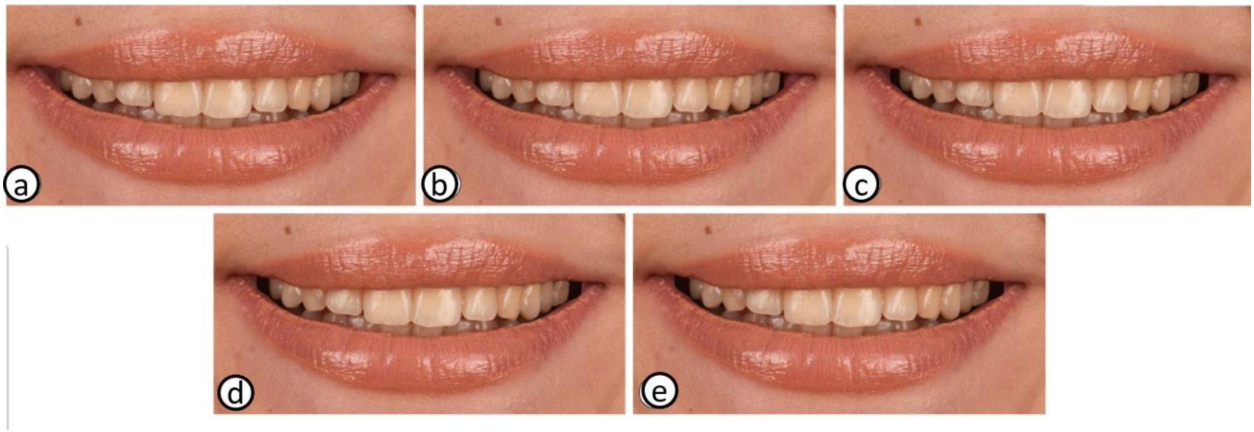

11. Photo-5 *

Mark only one option for each line.

	Very beautiful	Beautiful	Undecided	Ugly	Very ugly
a					
b					
c					
d					
e					

Photo-6

Photo 6

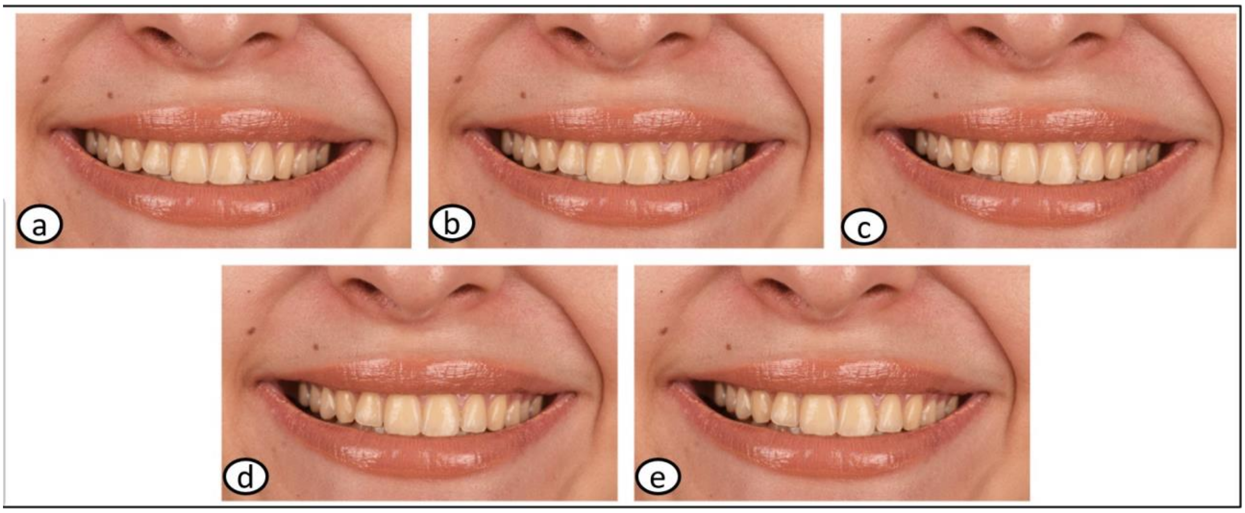

12. Photo-6 *

Mark only one option for each line.

	Very beautiful	Beautiful	Undecided	Ugly	Very ugly
a	◯	◯	◯	◯	◯
b	◯	◯	◯	◯	◯
c	◯	◯	◯	◯	◯
d	◯	◯	◯	◯	◯
e	◯	◯	◯	◯	◯

Photo-7

Photo 7

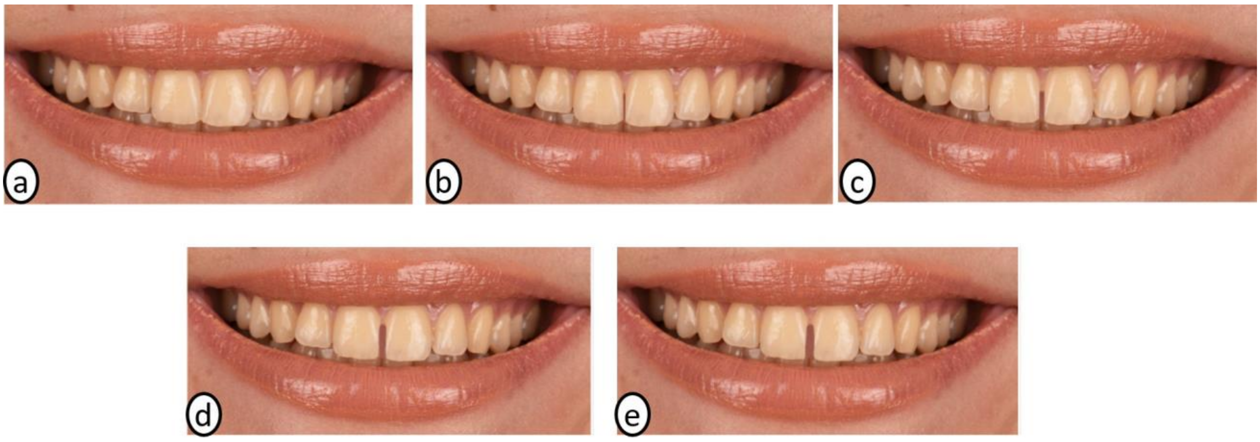

13. Photo-7 *

Mark only one option for each line.

	Very beautiful	Beautiful	Undecided	Ugly	Very ugly
a					
b					
c					
d					
e					

Photo-9

Photo-9

Photo 8

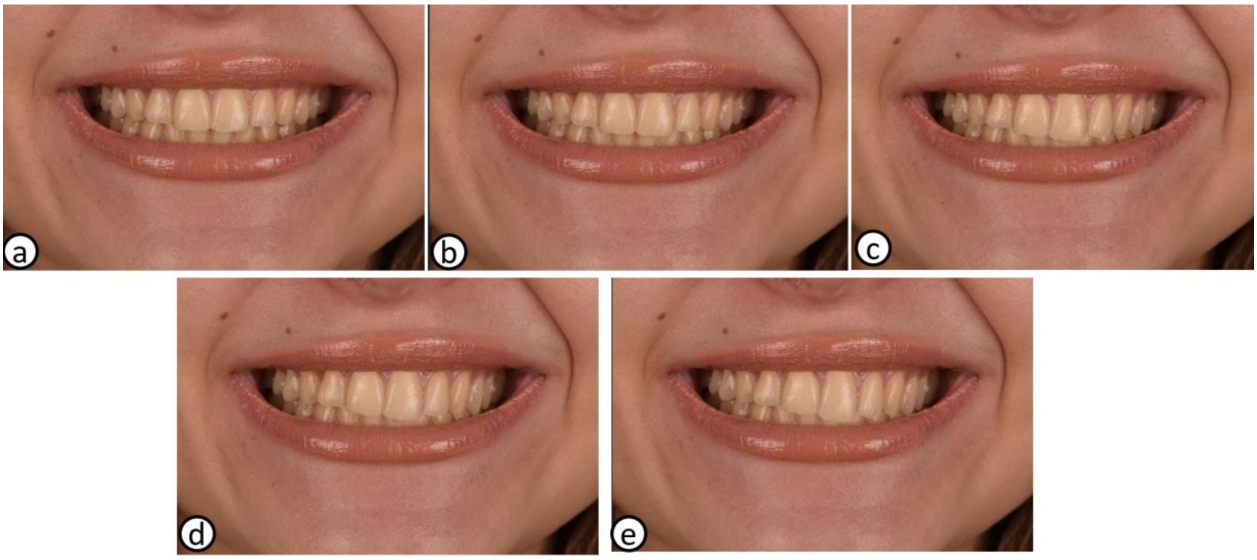

14. Photo-8 *

Mark only one option for each line.

	Very beautiful	Beautiful	Undecided	Ugly	Very ugly
a					
b					
c					
d					
e					

Photo-10

Photo 9

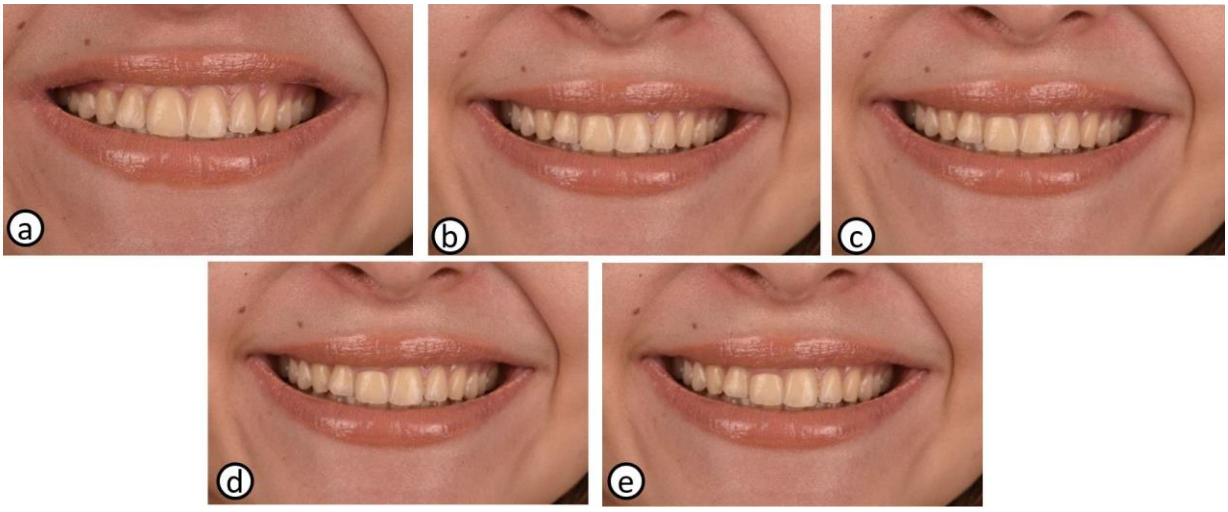

15. Photo-9 *

Mark only one option for each line.

	Very beautiful	Beautiful	Undecided	Ugly	Very ugly
a					
b					
c					
d					
e					

Photo-10

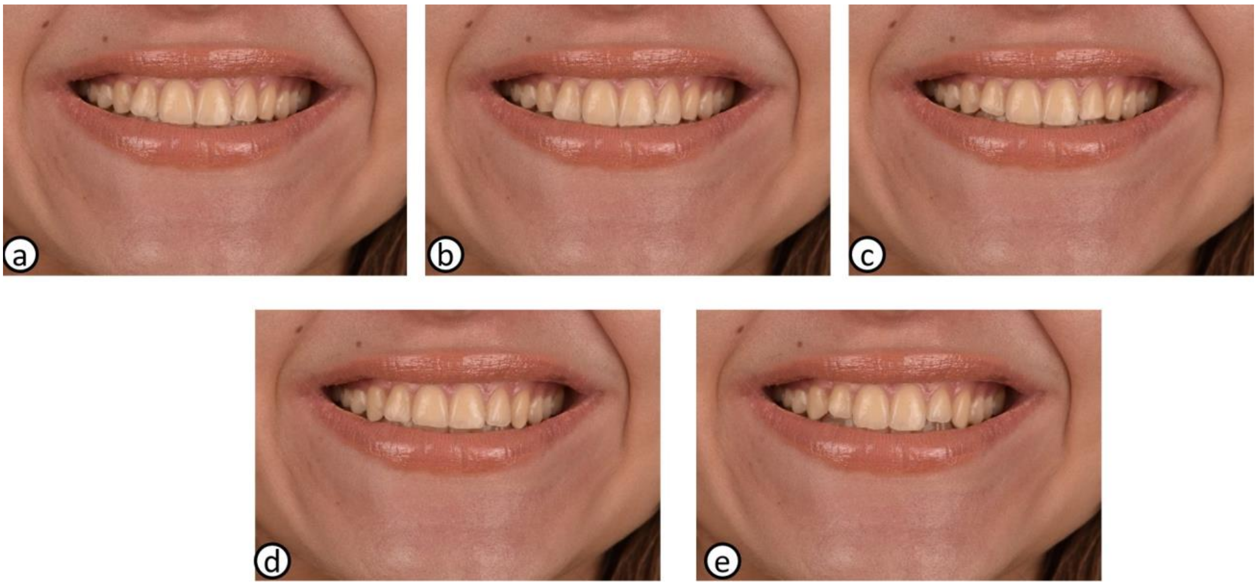

16. Photo-10 *

Mark only one option for each line.

	Very beautiful	Beautiful	Undecided	Ugly	Very ugly
a					
b					
c					
d					
e					

Bu içerik Google tarafından oluşturulmamış veya onaylanmamıştır.
