# Supplementary material for: Assessment of dental students’ perceptions of facial and smile aesthetics: impact of gender, education level, and family background
Source: BMC Med Educ. 2025 Oct 3;25:1350. doi: 10.1186/s12909-025-07931-z (PMC12495680; doi:10.1186/s12909-025-07931-z)
Supplement: Supplementary file 2 — Supplementary Material 2. [file 12909_2025_7931_MOESM2_ESM.pdf]

T.C.
İSTANBUL KENT ÜNİVERSİTESİ
Sağlık Bilimleri Bilimsel Araştırma ve Yayın Etiği Kurulu

Sayı : E-10420511-050-29360 17.01.2024
Konu : Etik Kurul Onayı

Sayın Dr. Öğretim Üyesi Pınar ŞEŞEN

Yürütücülüğü yapacağınız "Farklı Klinik Eğitim Derecelerine Sahip Diş Hekimliği Öğrencilerinin Gülüş Estetiğini Etkileyen Faktörleri Algılaması-Bir Anket Çalışması" çalışmanız ile ilgili başvurunuz Üniversitemiz Sağlık Bilimleri Araştırma ve Yayın Etiği Kurulu'nun 17.01.2024 tarihli, 2024-01 sayılı toplantısında görüşülerek ekteki şekliyle uygun olduğuna karar verildi.

Bilgilerinizi rica ederim.

Prof. Dr. Ahmet Tuğrul BİREN
Başkan

Ek: Raportör Değerlendirme Formu

Adres: Cihangir Mahallesi Sıraselviler Caddesi No:71 Beyoğlu İstanbul
Telefon No : 2126101010 Fax No : 2122445368
e-Posta : saglikbilimleri.ayek@kent.edu.tr İnternet Adresi : https://www.kent.edu.tr
Kep Adresi : istanbulkentuniversitesi@hs03.kep.tr
Bilgi İçin :Bahar TURGUT
Fakülte Sekreteri
Dahili No:371
