# Supplementary material for: Assessment of dental students’ perceptions of facial and smile aesthetics: impact of gender, education level, and family background
Source: BMC Med Educ. 2025 Oct 3;25:1350. doi: 10.1186/s12909-025-07931-z (PMC12495680; doi:10.1186/s12909-025-07931-z)
Supplement: Supplementary file 3 — Supplementary Material 3. [file 12909_2025_7931_MOESM3_ESM.pdf]

İSTANBUL KENT ÜNİVERSİTESİ

İSTANBUL KENT ÜNİVERSİTESİ
ETİK KURUL BAŞKANLIĞI
ARAŞTIRMA RAPORTÖRÜ DEĞERLENDİRME FORMU

Protokol No:

YIL__2024__/No_01

(Araştırmacı: Dotor Öğretim Üyesi Pınar Şeşen.......................................)

Araştırmanın Adı:

..."Farklı Klinik Eğitim Derecelerine Sahip Diş Hekimliği Öğrencilerinin Gülüş Estetiğini Etkileyen Faktörleri Algılaması- Bir Anket Çalışması..
....
....

Karar:

A) Etik Kurul onayını alır.

B) Eksikleri var, tamamlandığı takdirde kabul edilir.

C) Eksikleri var, tamamlandığı takdirde tekrar incelenir.

D) Ret

Gerekçesi:

Öneriler:

Araştırma Raportörünün Adı, Soyadı: : Prof. Dr.Fatma Ünalan

Tarih 08 /01 / 2024):

İmzası:

FÜnalan

Cihangir Mahallesi, Sıraselviler Caddesi, No:71, 34433 Beyoğlu İSTANBUL

İSTANBUL KENT ÜNİVERSİTESİ

İSTANBUL KENT ÜNİVERSİTESİ
ETİK KURUL BAŞKANLIĞI
ARAŞTIRMA RAPORTÖRÜ DEĞERLENDİRME FORMU

ARAŞTIRMA RAPORTÖRÜ KONTROL LİSTESİ

PROTOKOL NO: 24 /01

**GİRİŞ**	**EVET**	**HAYIR**	**AÇIKLAMA**
Araştırma proje konusu ile ilgili alanda ulusal ve uluslararası literatür taranarak, bir literatür analizi ile birlikte verilmiş midir?	+		
**AMAÇ**			
Çalışmanın Amaçları (birincil amaçlar, varsa ikincil, üçüncül, vs. amaçlar) belirlenmiş midir?	+		
**GEREÇ ve YÖNTEM**			
Araştırma bölgesi ve toplumu tanımlanmış mıdır?	+		
Varsa araştırma ile ilgili hipotezler tanımlanmış mıdır?	+		
Varsa özel terimler ayrıntılı olarak tanımlanmış mıdır?	+		
Karşılaştırılacak hasta/kontrol grup sayıları belirlenmiş midir?	+		
Varsa Kontrol grup/grupları tanımlanmış mıdır?	YOK		
Araştırmaya dâhil etme kriterleri belirlenmiş midir?	+		
Araştırmadan dışlanma kriterleri belirlenmiş midir?	+		
Araştırmadan çıkarılma kriterleri belirlenmiş midir?	+		
Araştırma için toplanması gerekli veriler (değişkenler) tanımlanmış mıdır?	+		
Veri Toplama Yöntemi kararlaştırılmış mıdır? (Anket/Kayıt taraması/Fiziksel ve/veya Laboratuvar Muayene)	+		
Verilerin ölçüm biçimleri (kullanılacak ölçüm yöntemi/niceliksel ya da niteliksel karakterde olup olmaması/niceliksel ölçüm sonuçlarının beklenen minimum-maksimum değerleri / niteliksel ölçüm sonuçlarının kategorileri) belirlenmiş midir?	+		
Veri toplamak için kullanılacak cihazların marka ve modeli tanımlanmış mıdır?	+		
Veri derlemek için kullanılacak ölçeklerin geçerlik ve güvenirlik özellikleri literatüre dayanılarak tanımlanmış mıdır?		+	
Veri toplama aşamasında hizmet alımı kullanılacak mıdır?		+	
Varsa Hizmet alımının nasıl yapılacağı tanımlanmış mıdır?		+	
Uygulamayı kimin yapacağı, nasıl seçileceği ve eğitim verileceği tanımlanmış mıdır?	+		
Varsa Körleme yöntemi tanımlanmış mıdır?		YOK	
Varsa Rassallık (Randomizasyon) yöntemi tanımlanmış mıdır?		YOK	
Varsa Eşleştirme (Matching) yöntemi tanımlanmış mıdır?		YOK	
Varsa araştırmada görev alacak yardımcı personelin seçimi ve eğitimi belirlenmiş midir?		YOK	
Veri derleme öncesinde Ön Uygulamanın yapılıp yapılmayacağı belirtilmiş midir?	+		
Araştırma tasarımı zaman çizelgesine uygun olarak tanımlanmış mıdır?	+		
Örneklem büyüklüğü ve örnekleme yöntemi tanımlanmış mıdır?		+	
Kullanılacak olan istatistiksel yöntemler açık bir şekilde tanımlanmış mıdır?		+	
**GÖNÜLLÜ BİLGİLENDİRİLMİŞ OLUR FORMU EKLENMİŞ Mİ?**	+		
**DESTEKLEYİCİ BİLGİLERİ BELİRTİLMİŞ Mİ?**		YOK	
**GEREKLİ İSE SİGORTA TEMİNATI YAPILMIŞ MI?**		YOK	

Araştırma Raportörünün Adı, Soyadı: Prof. Dr.Fatma Ünalan

Tarih (08 / 01 / 2024): **İmzası:** [signature]
